# Myoclonic Jerks and Schizophreniform Syndrome: Case Report and Literature Review

**DOI:** 10.3389/fpsyt.2018.00161

**Published:** 2018-05-01

**Authors:** Dominique Endres, Dirk-M. Altenmüller, Bernd Feige, Simon J. Maier, Kathrin Nickel, Sabine Hellwig, Jördis Rausch, Christiane Ziegler, Katharina Domschke, John P. Doerr, Karl Egger, Ludger Tebartz van Elst

**Affiliations:** ^1^Section for Experimental Neuropsychiatry, Department of Psychiatry and Psychotherapy, Medical Center — University of Freiburg, Faculty of Medicine, University of Freiburg, Freiburg, Germany; ^2^Department of Psychiatry and Psychotherapy, Medical Center — University of Freiburg, Faculty of Medicine, University of Freiburg, Freiburg, Germany; ^3^Department of Neurosurgery, Medical Center — University of Freiburg, Faculty of Medicine, University of Freiburg, Freiburg, Germany; ^4^Department of Neuroradiology, Medical Center — University of Freiburg, Faculty of Medicine, University of Freiburg, Freiburg, Germany

**Keywords:** juvenile myoclonic epilepsy, myoclonic jerks, Janz syndrome, schizophrenia, paraepileptic, LANI-hypothesis

## Abstract

**Background:** Schizophreniform syndromes can be divided into primary idiopathic forms as well as different secondary organic subgroups (e.g., paraepileptic, epileptic, immunological, or degenerative). Secondary epileptic explanatory approaches have often been discussed in the past, due to the high rates of electroencephalography (EEG) alterations in patients with schizophrenia. In particular, temporal lobe epilepsy is known to be associated with schizophreniform symptoms in well-described constellations. In the literature, juvenile myoclonic epilepsy has been linked to emotionally unstable personality traits, depression, anxiety, and executive dysfunction; however, the association with schizophrenia is largely unclear.

**Case presentation:** We present the case of a 28-year-old male student suffering from mild myoclonic jerks, mainly of the upper limbs, as well as a predominant paranoid-hallucinatory syndrome with attention deficits, problems with working memory, depressive-flat mood, reduced energy, fast stimulus satiation, delusional and audible thoughts, tactile hallucinations, thought inspirations, and severe sleep disturbances. Cerebral magnetic resonance imaging and cerebrospinal fluid analyses revealed no relevant abnormalities. The routine EEG and the first EEG after sleep deprivation (under treatment with oxazepam) also returned normal findings. Video telemetry over one night, which included a partial sleep-deprivation EEG, displayed short generalized spike-wave complexes and polyspikes, associated with myoclonic jerks, after waking in the morning. Video-EEG monitoring over 5 days showed over 100 myoclonic jerks of the upper limbs, frequently with generalized spike-wave complexes with left or right accentuation. Therefore, we diagnosed juvenile myoclonic epilepsy.

**Discussion:** This case report illustrates the importance of extended EEG diagnostics in patients with schizophreniform syndromes and myoclonic jerks. The schizophreniform symptoms in the framework of epileptiform EEG activity can be interpreted as a (para)epileptic mechanism due to local area network inhibition (LANI). Following the LANI hypothesis, paranoid hallucinatory symptoms are not due to primary excitatory activity (as myoclonic jerks are) but rather to the secondary process of hyperinhibition triggered by epileptic activity. Identifying subgroups of schizophreniform patients with comorbid epilepsy is important because of the potential benefits of optimized pharmacological treatment.

## Background

Schizophreniform syndromes are characterized by delusions, hallucinations, thought disorders, cognitive impairment, and social withdrawal (1). Besides the primary, idiopathic, and polygenetic forms, different secondary pathophysiological mechanisms (e.g., immunological, degenerative, monogenetic, metabolic, epileptic, or paraepileptic) can be assumed (2, 3). Epileptic or paraepileptic explanatory approaches have traditionally been used due to the high rates of electroencephalography (EEG) alterations in patients with schizophreniform syndromes (4–8). In line with this assumption, we have reported the case of a young patient with a schizophreniform syndrome and generalized spike-wave complexes, but without seizures. This patient achieved full psychotic remission under treatment with valproate (9, 10). More recently, we have also reported a case of a female patient with schizophreniform syndrome and generalized 3 Hz polyspike wave complexes, but without seizures. This patient reached complete remission under treatment with levetiracetam (11). While a pathogenetic link between temporal lobe epilepsy and schizophreniform symptoms has been extensively discussed in the literature (12)—an observation that had led to the temporal lobe hypothesis for schizophrenia (13, 14)—a pathophysiological connection between primary generalized forms of epilepsy and schizophrenia is rarely proposed (7).

The association between juvenile myoclonic epilepsy (also called Janz syndrome) and schizophreniform syndromes is largely unclear. Janz syndrome is a frequent, age-related, and inheritable disorder with prevalence estimated to be 5–10% of all epilepsies and approximately 18% of idiopathic generalized epilepsies (15). It is characterized by seizures with bilateral, arrhythmic myoclonic jerks, mainly of the arms, without disturbance of consciousness (16). Psychiatric symptoms include emotionally unstable personality traits with rapid mood changes, depression, anxiety, substance abuse, and executive dysfunction (17, 16). Often, generalized tonic-clonic seizures can be observed; their absence is less frequent. In addition, about a third of patients with Janz syndrome have typical absence seizures. Myoclonic jerks typically occur after awakening and are pronounced after sleep deprivation. In the interictal and ictal EEG, generalized spike-wave complexes and polyspikes waves are commonly found (16).

## Case presentation

We present the case of a 28-year-old male student who had suffered from fluctuating paranoid-hallucinatory symptoms over the last seven years (since the age of 21 years) and who had been psychiatrically hospitalized five times due to this condition. On first admission to our department at the age of 25, the patient reported increasing problems, with difficulties in studying and social withdrawal, over the past several weeks. He reported attention and concentration deficits, as well as problems with “working memory.” Formal thought processes were slowed, the mood was depressive-flat, and energy was decreased. The patient displayed suspicious behavior and sensory overload phenomena. Moreover, he reported ideas of reference and delusional thoughts thinking somebody might poison him. Other psychotic features included dysmorphic delusions, acouasms, thought reading, broadcasting and insertions plus tactile hallucinations. The patient also reported severe sleep disturbances with sleep-onset insomnia and a sleep phase delay, as well as suicidal thoughts. Approximately three years (at the age of 24 years) after the beginning of paranoid-hallucinatory symptoms, the patient developed myoclonic jerks. During the first occurrence of myoclonic jerks, the patient was treated with fluoxetine and zopiclone. Before onset of first myoclonic jerks the patient was already treated with different neuroleptics (aripiprazole, olanzapine, promethazine, quetiapine), as well as the antidepressant duloxetine and the benzodiazepine oxazepam. All these neuroleptics and duloxetine were not tolerated and only prescribed for short time. The myoclonic jerks mostly affected the right arm, but sometimes also the left arm and occasionally the legs. He described “electric shock feelings” in his body while having the myoclonic jerks. At the age of 26, the patient had one isolated bilateral tonic-clonic seizure (during the tapering of oxazepam). Treatment with different neuroleptics led to an increase of the myoclonic jerks. Sleep deprivation also led to an increase in myoclonic jerks. In addition, they were specifically triggered by writing.

### Developmental, somatic, and family history

The patient's developmental history was negative for *in utero* or birth complications, febrile convulsions, and inflammatory brain diseases. At the age of 11, he experienced a mild cerebral contusion. In primary school, he was affected by symptoms of attention deficit hyperactivity disorder. No autistic features or tic symptoms were reported. At the age of 14, he had problems at school and abused cannabis and alcohol. After graduating from high school, he started studying economics. His medical history revealed only bronchial hyper-reactivity. The family history was negative for myoclonic jerks or epilepsy; however, the mother was described as having an emotionally unstable personality.

### Investigations

**Figure 1:**
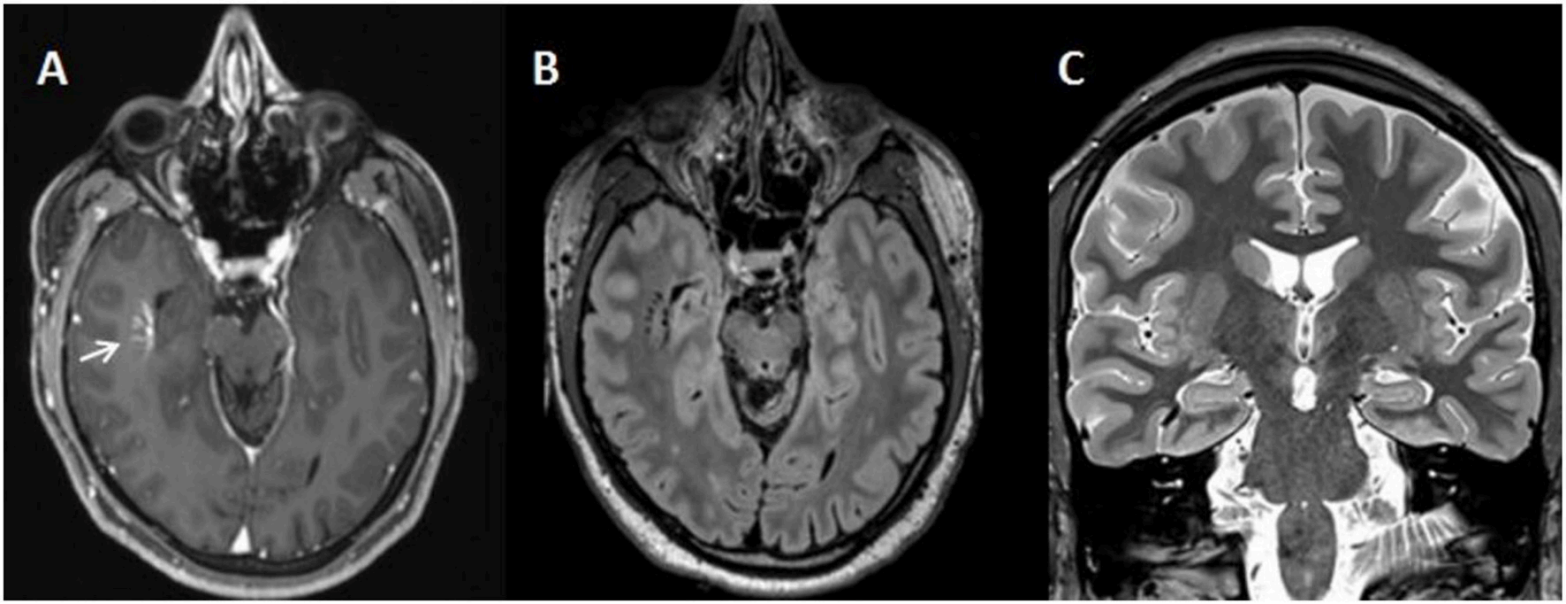
Cerebral magnetic resonance imaging (cMRI) showing an isolated uncomplicated developmental venous anomaly in the right temporal lobe (white arrow) on the contrast enhanced initial axial MPRAGE **(A)** and on the 2 year follow-up epilepsy specific high-resolution T2-weighted axial reformatted 3D-FLAIR **(B)** and coronal T2-weighted STIR **(C)** images. cMRI scans were acquired on a Siemens 3T scanner TRIO **(A)** and PRISMA **(B,C)**.

**Figure 2:**
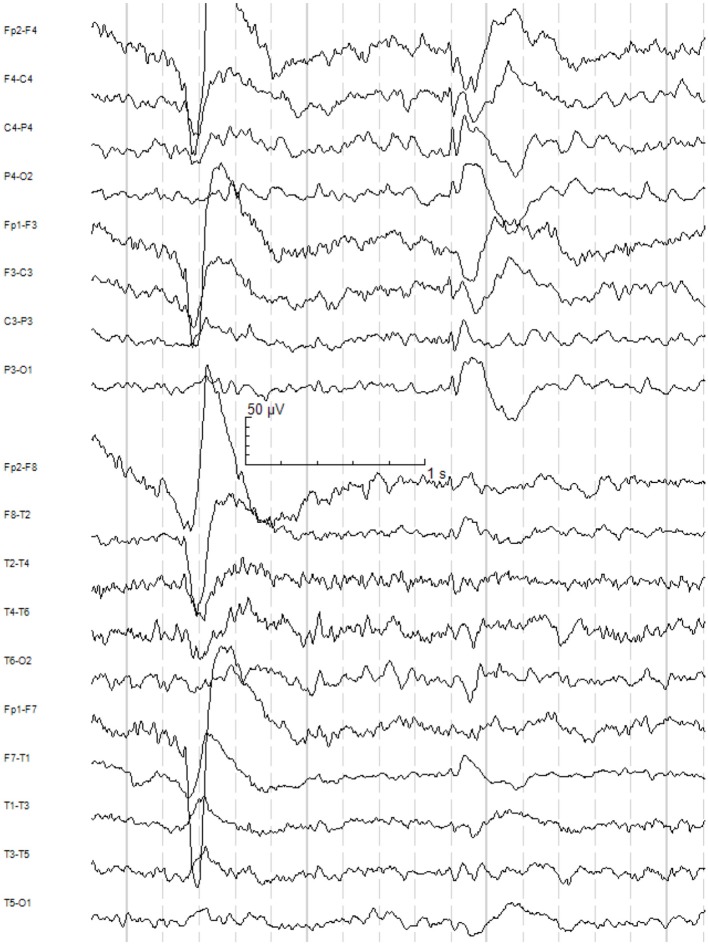
Ictal EEG recording during long-term video-EEG monitoring: Generalized spike-wave complex with fronto-central maximum associated with a habitual myoclonic jerk of the right hand during writing. Longitudinal bipolar montage, 50 Hz notch filter, low-pass filter 30 Hz, high-pass filter 1 Hz.

Most of the investigations were performed during the patient's first stay in our department when he was 25. Blood analyses showed a vitamin D deficiency; renal, liver, and thyroid functions were normal. Thyroid autoantibodies (*against thyroglobulin, thyroid peroxidase, and thyroid-stimulating hormone*), antibodies against intracellular onconeural antigens (*Yo, Hu, CV2/CRMP5, Ri, Ma1/2, SOX1*), and intracellular synaptic antigens (*GAD, amphiphysin*) showed no abnormalities. Cerebrospinal fluid (CSF) analyses were essentially normal with a regular white cell count (1 μL; reference <5 μL), no blood-brain barrier dysfunction (protein concentration: 341; reference <450 mg/L; albumin quotient of 4; age-dependent reference <6.5 × 10^−3^), and no oligoclonal bands. CSF antibodies against neuronal cell surface antigens (*NMDAR, AMPA-R, GABA-B-R, VGKC-complex [LGI1, Caspr2]*) were negative. The initial contrast-enhanced cerebral magnetic resonance imaging (cMRI) as well as the 2-year follow-up epilepsy-specific cMRI including high-resolution 2D- and isotropic 3D-MRI-sequences showed an isolated and uncomplicated developmental venous anomaly (DVA) in the right temporal lobe (Figure 1). There was no associated epileptogenic lesion, such as cavernoma or cortical dysplasia. Taken together, we did not find any evidence of an immunological encephalopathy or other relevant inflammatory diseases. The routine EEG was normal. The first EEG after sleep deprivation also showed no slow or epileptic activity; however, the patient had been treated with oxazepam at that time. Therefore, we performed a 24h video telemetry (under treatment with amisulpride 600 mg and escitalopram 10 mg) including a partial sleep EEG, in combination with measurement of melatonin levels. During this measurement, the patient was awake until 3:00 a.m. Subsequently, he slept for 5 h. During the whole period, the patient reported three mild myoclonic jerks of the right arm. These myoclonic jerks were not associated with epileptic activity in the EEG. However, in the following period, after awakening in the morning, the patient reported six myoclonic jerks of the right arm. All of these presented with simultaneous epileptic activity in the EEG characterized by brief (<3 s) trains of generalized polyspikes and spike-wave complexes with left fronto-central maximum. For syndrome diagnosis, additional long-term video-EEG monitoring (under treatment with clozapine 75 mg, escitalopram 20 mg, oxcarbazepine gradually reduced, and brivaracetam stopped) was carried out when the patient was 27. The monitoring was performed over 5 days and, during this period, the patient showed over 100 myoclonic jerks of the upper limbs (right, left, and bilaterally synchronous), frequently associated with generalized spike-wave complexes (Figure 2) and occasionally with left or right accentuation. All clinical events were praxis-induced (by writing with the right or left hand). Additional absences or bilateral tonic-clonic seizures were not documented. The interictal EEG showed frequent intermittent generalized theta slowing, but no clear-cut epileptiform activity. Intermittent photic stimulation did not evoke photoparoxysmal responses.

### Differential diagnosis

In light of the ictal generalized epileptiform activity, the seizure semiology characterized by praxis-induced myoclonic jerks of both arms with worsening after sleep deprivation, and the history of one additional bilateral tonic-clonic seizure, we diagnosed juvenile myoclonic epilepsy (Janz syndrome). The generalized EEG activity, the absence of any potentially epileptogenic lesion in the cMRI, and the semiology with myoclonic jerks of both arms suggested that the epilepsy was not focal. Psychotropic drugs might have triggered or strengthened the symptoms due to genetic vulnerability; however, it is unlikely that the persistent myoclonic jerks are only a side effect of psychotropic drugs because myoclonic jerks also occurred over longer periods with solely antiepileptic treatment with carbamazepine. Myoclonic jerks could also be due to paraneoplastic or non-paraneoplastic inflammatory processes such as immunological encephalopathies or opsoclonus-myoclonus syndrome. In our case, the normal cMRI and CSF findings speak against this idea. Normal copper and ceruloplasmin levels and the normal cMRI findings are not compatible with Morbus Wilson. We also found no neurological signs of progressive myoclonus epilepsies such as Unverricht-Lundborg disease or Lafora disease. Clinically, there were no signs (e.g., labyrinthine deafness, optic atrophy, or myopathy), pointing to a mitochondrial myoclonic epilepsy (e.g., with ragged red fibers or MERRF syndrome). However, a new mitochondrial disease cannot be completely ruled out. The extensive investigation was performed because of the atypical neuropsychiatric presentation with paranoid hallucinatory symptoms and myoclonic jerks. The psychiatric symptoms formally fulfilled the criteria of paranoid-hallucinatory schizophrenia following the International Statistical Classification of Diseases and Related Health Problems (10th revision; ICD-10). Due to the temporal association regarding the development of myoclonic jerks and schizophreniform symptoms, as well as the partial improvement of the psychiatric symptoms with antiepileptic treatment, we suspected a common (para-)epileptic pathomechanism. Alternatively, the presence of Janz syndrome can be a simple comorbidity in primary schizophrenia.

### Therapy and outcome

Initially, the patient was treated unsuccessfully with different antidepressants (bupropion, duloxetine, fluoxetine, mirtazapine, and venlafaxine) and neuroleptics (amisulpride, aripiprazole, olanzapine, promethazine, quetiapine, and risperidone). During treatment with amisulpride, quetiapine, and risperidone, an increased rate of myoclonic jerks was reported. The treatment of early dyskinesia with biperiden led to a dramatic increase in the myoclonic jerks. Oxazepam quickly reduced the rate of myoclonic jerks and was taken by the patient over a longer period. Promethazine also led to an increased rate of myoclonic jerks.

Since his first stay in our department, when the patient was 25, we have prescribed various anticonvulsants. However, many antiepileptic drugs were not tolerated (valproate due to an increase of pancreatic enzymes, topiramate due to subjectively experienced cognitive deficits, levetiracetam due to daily tiredness, and perampanel due to dizziness and daily tiredness). Of these, topiramate led to a rapid reduction of myoclonic jerks, even at low doses of 25 mg; however, psychiatric symptoms persisted. Under the sole treatment with carbamazepine (up to 900 mg), the myoclonic jerks and paranoid hallucinatory symptoms temporarily improved; however, they did not disappear completely and ongoing. Zonisamide (up to 100 mg) was tolerated but had no relevant positive effects. Oxazepam (different doses) and clobazam (up to 20 mg) led to the reduction of myoclonic jerks. Oxcarbazepine (up to 2,400 mg) was well tolerated, though its effect was unclear. In combination with brivaracetam (200–250 mg), the myoclonic jerks were reduced in frequency and severity.

Over one year, the patient was treated with clozapine (75 mg), brivaracetam (200–250 mg), and oxcarbazepine (2,400 mg). Clozapine led to a reduction in delusions, hallucinations, and thought inspirations, though these psychotic symptoms did not disappear completely. Higher doses were not tolerated due to an increased frequency of myoclonic jerks. A hypochondriac personality type, together with delusions and dysmorphophobic tendencies, persisted as well as mood fluctuations and concentration deficits. Furthermore, the patient still had about 10 writing-induced myoclonic jerks per day.

## Discussion

We presented the case of a patient with paranoid-hallucinatory syndrome in the context of juvenile myoclonic epilepsy.

### Previous findings

In a retrospective study analyzing 100 patients with Janz syndrome, nearly 50% suffered from Axis I psychiatric disorders. Anxiety (23%) and mood disorders (19%) were most frequent, while 7% suffered from somatoform disorders and three cases (3%) were reported to have schizophrenia. The temporal association between the occurrence of myoclonic jerks and schizophrenia was not reported. Seventeen cases fulfilled the “Structured Clinical Interview for DSM Disorders” (SCID) II criteria for cluster B personalities (histrionic, borderline, and passive-aggressive) (17). In an older study of 170 patients, only 26.5% suffered from a comorbid psychiatric disorder. Psychotic disorders were found in 2.9%; however, only one case (0.6%) fulfilled criteria for paranoid-hallucinatory schizophrenia. Depressive disorders (1.8%) and anxiety disorders (3.5%) were less frequent in this study. Personality disorders were found in 14.1% of cases, mainly with BPD (18). In the study from Somayajula et al., analyzing 165 patients with juvenile myoclonic epilepsy in India, 47% were diagnosed with psychiatric disorders, which were mostly anxiety disorders (30.4%) and depression (15.7%). Only one patient (0.6%) suffered from schizophrenia (19). In summary, schizophrenia seems to be infrequent in Janz syndrome. The individual cases of patients with Janz syndrome and schizophrenia, reported in the larger studies mentioned above, are not presented in detail. No case reports are available analyzing this important association. Therefore, in our opinion, case studies like the present one are necessary.

### Diagnostic process

After the initial normal routine and sleep-deprivation EEG, one could have speculated that the myoclonic jerks were merely psychogenic especially because the patient described them as “electric shocks” in his body. However, patients with Janz syndrome often describe the seizures as comparable with “electric shocks.” Therefore, this syndrome often has a meaningful delay in the diagnosis because myoclonic jerks are not recognized. Usually patients seek for a medical consultation only after the first generalized-tonic clonic seizure. Therefore, directly asking the patients about myoclonic seizures is a key procedure for diagnosis. Normal interictal EEGs are possible in patients with Janz syndrome. Therefore, a detailed anamnesis and extended EEG analyses including video-telemetry may help in correct diagnostic identification, as we did in our patient.

### Pathophysiological interpretation

Schizophreniform syndromes and epilepsy may share common genetic susceptibility (20, 21). The LANI hypothesis could explain a potential causal relationship between epileptiform EEG activity, myoclonic jerks, and schizophreniform syndromes. The genetically determined and pathologically enhanced excitatory network activity seen in the surface EEG in the form of generalized epileptiform potentials presenting clinically with (praxis-induced) myoclonic seizures and a bilateral tonic-clonic seizure might lead to consecutive inhibitory processes in a homoeostatic, electrophysiological attempt to stabilize cerebral networks. In our patient, we found recurrent excitatory activity (primarily epileptiform activity, but also interictal intermittent generalized theta slowing) that could have exceeded a critical threshold, leading to successive hyperinhibition of cerebral networks. Following this theory, schizophreniform symptoms would not be due to primary excitatory activity as represented by myoclonic jerks, but to the secondary homoeostatic process of hyperinhibition. Frontal hyperinhibition might have led to attention and concentration deficits, and temporal hyperinhibition might have led to hallucinations or memory deficits (7, 10). The LANI hypothesis allows an explanation of short-term schizophreniform symptoms in the present patient. It needs to be further investigated using animal studies, as well as imaging and electrophysiological measurements. Longer-lasting personality traits and cognitive deficits in patients with Janz syndrome might alternatively be interpreted as a form of an epileptic encephalopathy (22, 23).

### Treatment strategies

Identifying schizophreniform subgroups of patients with such (para-)epileptic pathomechanisms may allow new treatment options with antiepileptics for these individuals. For patients with Janz syndrome, treatment alternatives include valproate, lamotrigine, levetiracetam, topiramate, or zonisamide (24). All these substances were tried in our patient except for lamotrigine, which can sometimes worsen myoclonic jerks (24) and which needs a long period for dosage increase. Carbamazepine and oxcarbazepine are also known to aggravate myoclonic jerks (24); however, this was not the case in our patient who showed temporary improvement especially under treatment with carbamazepine. Interestingly, the antiepileptics valproate and lamotrigine are already approved for augmentative therapy in schizophrenia (25–27). It can be assumed that patients with (para)epileptic pathomechanisms and comorbidity may benefit more from such anticonvulsive augmentation strategies than other subgroups (e.g., polygenetic subtypes). Moreover, it is important to recognize that different neuroleptics and biperiden can reduce the seizure threshold (7) and, therefore, may push this pathophysiology and worsen myoclonic jerks in patients with a relevant predisposition (especially because the first myoclonic jerks occurred at first after treatment with psychotropic drugs in our case report patient).

This reaction was the reason for the intolerance of neuroleptic monotherapy in the history of our patient. Moreover, following the general assumptions of the LANI model, the improvement that followed a low dose of clozapine treatment can be explained by its proconvulsive properties, which might have overcome hyperinhibitory network states, although it is likely to improve the causative excitatory activity.

## Conclusion

This case report shows the importance of the clinical history in patients with myoclonic jerks, which should be taken from the patients and at least one person who witnessed the seizures. Moreover, it shows that EEG diagnostic is very useful in psychiatric patients. In unclear cases with clinical suspicion of mild Janz syndrome with predominant psychiatric manifestations, a sleep-deprivation EEG and long-term video-EEG monitoring may help with the diagnosis of Janz syndrome in the case of normal routine EEG. Identifying subgroups of schizophreniform patients with (para)epileptic pathomechanisms is important because these patients might improve under antiepileptic treatment and might deteriorate under isolated neuroleptic medication.

## Consent for publication

The patient has given his informed and written consent for this case report, including the presented images, to be published.

## Author contributions

DE, D-MA and LTvE treated the patient. DE wrote the Paper. All authors were crucial involved in the theoretical discussion and the preparation of the manuscript. All authors read and approved the final version of the manuscript.

### Conflict of interest statement

D-MA: Lecture fees from UCB Pharma; LTvE: Lectures, workshops or travel grants within the last 3 years: Eli Lilly, Medice, Shire, UCB, Servier, and Cyberonics. DE, BF, SM, KN, SH, JR, CZ, KD, JD, and KE declare that the research was conducted in the absence of any commercial or financial relationships that could be construed as a potential conflict of interest.
